# Expression, covariation, and genetic regulation of miRNA Biogenesis genes in brain supports their role in addiction, psychiatric disorders, and disease

**DOI:** 10.3389/fgene.2013.00126

**Published:** 2013-07-05

**Authors:** Megan K. Mulligan, Candice DuBose, Junming Yue, Michael F. Miles, Lu Lu, Kristin M. Hamre

**Affiliations:** ^1^Department of Anatomy and Neurobiology, University of Tennessee Health Science CenterMemphis, TN, USA; ^2^Department of Pathology, University of Tennessee Health Science CenterMemphis, TN, USA; ^3^Departments of Pharmacology/Toxicology and Neurology, Virginia Commonwealth UniversityRichmond, VA, USA; ^4^Jiangsu Key Laboratory of Neuroregeneration, Nantong UniversityNantong, China

**Keywords:** BXD, miRNA biogenesis, addiction, drosha, dicer1, Dgcr8, disease

## Abstract

The role of miRNA and miRNA biogenesis genes in the adult brain is just beginning to be explored. In this study we have performed a comprehensive analysis of the expression, genetic regulation, and co-expression of major components of the miRNA biogenesis pathway using human and mouse data sets and resources available on the GeneNetwork web site (genenetwork.org). We found a wide range of variation in expression in both species for key components of the pathway—*Drosha, Pasha*, and *Dicer*. Across species, tissues, and expression platforms all three genes are generally well-correlated. No single genetic locus exerts a strong and consistent influence on the expression of these key genes across murine brain regions. However, in mouse striatum, many members of the miRNA pathway are correlated—including *Dicer, Drosha, Pasha, Ars2 (Srrt*), *Eif2c1* (*Ago1*), *Eif2c2* (*Ago2*), *Zcchc11*, and *Snip1*. The expression of these genes may be partly influenced by a locus on Chromosome 9 (105.67–106.32 Mb). We explored ~1500 brain phenotypes available for the C57BL/6J × DBA/2J (BXD) genetic mouse population in order to identify miRNA biogenesis genes correlated with traits related to addiction and psychiatric disorders. We found a significant association between expression of *Dicer* and *Drosha* in several brain regions and the response to many drugs of abuse, including ethanol, cocaine, and methamphetamine. Expression of *Dicer, Drosha*, and *Pasha* in most of the brain regions explored is strongly correlated with the expression of key members of the dopamine system. *Drosha, Pasha*, and *Dicer* expression is also correlated with the expression of behavioral traits measuring depression and sensorimotor gating, impulsivity, and anxiety, respectively. Our study provides a global survey of the expression and regulation of key miRNA biogenesis genes in brain and provides preliminary support for the involvement of these genes and their product miRNAs in addiction and psychiatric disease processes.

## Introduction

Small non-coding microRNAs (miRNAs) are a recently discovered class of post-transcriptional regulatory molecules that control gene expression. Genes involved in the biogenesis of these regulatory RNAs include Dicer (Dicer1), Drosha, and Dgcr8 (Pasha). As part of the canonical pathway, Drosha and Pasha process pri-miRNA into pre-miRNA, which is then transported into the cytoplasm via Exportin 5 (Bohnsack et al., 2004). Pasha identifies the complex stem-loop structure of pri-mRNA and recruits Drosha, resulting in cleavage and the generation of pre-miRNA (Denli et al., 2004). Once in the cytoplasm, pre-miRNA is processed into miRNA by Dicer, Argonaute (Ago) proteins, accessory proteins, and TRBP. The ribonuclease Dicer processes pre-miRNA into a ~21 nt miRNA duplex as part of the RNA-induced silencing complex (RISC) loading complex and the duplex is further unwound by various Ago RNA-binding proteins in order to produce mature ~21 nt single stranded miRNAs. Loading of miRNA into the RISC is facilitated by TRBP and target matching between mRNAs and miRNAs occurs in the RISC, which is composed of Ago proteins and accessory proteins. For an excellent review of the miRNA biogenesis pathway in brain, please see O'Carroll and Schaefer (2013). Several alternative and so-called non-canonical pathways for miRNA biogenesis also exist, including a Dicer-dependent pathway which functions independently of Drosha and Pasha (Berezikov et al., 2007; Babiarz et al., 2008), and a Dicer-independent pathway that is dependent on the actions of Drosha and Ago2 (Cheloufi et al., 2010). Loss of function of any of these genes will thus have a severe impact of the miRNA landscape and the post-transcriptional regulation of mRNA (Babiarz et al., 2011).

Many of the miRNA biogenesis genes are required very early in development both due to the essential role of miRNAs in these processes and because biogenesis genes function in multiple biological processes. For example, *Dicer* knock down results in early embryonic lethality with a concomitant lack of stem cell populations (Bernstein et al., 2003) likely due to the absence of key developmental miRNAs but also potentially due to defects in differentiation and maintenance of heterochromatin structure (Kanellopoulou et al., 2005). miRNA biogenesis genes also have important roles in neuronal development, survival, and maintenance (Hsu et al., 2012; McLoughlin et al., 2012). Because of the critical nature of these genes during development, it is perhaps unexpected that they would also play roles in the mature central nervous system (CNS). However, it has been shown that genetic variants in miRNA processing genes have been linked to schizophrenia (Zhang et al., 2012; Zhou et al., 2013), depression (He et al., 2012), and Huntington's disease (Lee et al., 2011). In addition, a recent study by Tapocik et al. (2009) identified Dicer as one of the possible candidate genes mediating differential morphine analgesia between C57BL/6 (B6) and DBA/2J (D2) mouse strains. Addiction, psychiatric disorders, and neurological disease are influenced by genetic factors and these initial studies suggest that miRNA and miRNA biogenesis associated genes may play a role in the genetic regulation of these disorders.

In the present study, we provide the first comprehensive analysis of variation in miRNA biogenesis gene expression across both mouse and human populations and investigate the potential behavioral and physiological consequences of this variation. First, we use a large family of genetically diverse strains derived from a cross between B6 and D2—the BXD family—to dissect sources and potential consequences of miRNA biogenesis gene variation. Currently the largest and most well-characterized genetic family of mice, the BXD set of strains has been profiled for ~1500 brain and behavioral phenotypes and the whole family is densely genotyped. This set of ~80 strains has also been extensively profiled for gene expression across 12 brain regions and sub-regions using a variety of platforms. Finally, we compare the expression of miRNA biogenesis genes in human brain from normal, Alzheimer's (AD), and Huntington's (HD) disease cases. This wealth of molecular, genetic, and phenotypic data is freely available at the GeneNetwork (www.genenetwork.org) web site. We leverage the information available at this excellent functional genomics resource to investigate the genetic regulation, expression, and coexpression networks of miRNA biogenesis genes and to dissect their contribution to disease, addiction and psychiatric-related behaviors.

## Methods

### GeneNetwork database descriptions

The BXD family includes ~80 recombinant inbred strains derived at three different time points from separate crosses between a female B6 and a male D2. The F1 progeny were subsequently intercrossed followed by inbreeding to fix parental genotypes at each locus. The first set (BXD1 through 32) was created in the late 1970s and the second set (BXD33 through BXD42) was generated in the early 1990s, both by Benjamin Taylor. The third set (BXD43 through 100) was created in the late 1990s and early 2000s at UTHSC (Peirce et al., 2004). The following BXD brain expression data sets were used in this analysis: Hippocampus Consortium M430v2 (Jun06) RMA (Overall et al., 2009), INIA Amygdala Cohort Affy MoGene 1.0 ST (Mar11) RMA, HBP Rosen Striatum M430V2 (Apr05) RMA Clean, VCU BXD PFC Sal M430 2.0 (Dec06) RMA, VCU BXD PFC EtOH M430 2.0 (Dec06) RMA, VCU BXD VTA Sal M430 2.0 (Jun09) RMA, VCU BXD VTA EtOH M430 2.0 (Jun09) RMA, VCU BXD NA Sal M430 2.0 (Oct07) RMA, and VCU BXD NA EtOH M430 2.0 (Oct07) RMA. All data sets have been standardized to a mean log2 expression level of 8 with a standard deviation of 2. Average log2 expression less than 6 is considered to be background noise. Detailed information for each data set is available at genenetwork.org.

The following human brain data sets were also included,“GSE5281 Human Brain Full Liang (Jul09) RMA,” “GSE15222 Human Brain Meyers (Apr09) RankInv”, and Harvard Brain Tissue Resource Center “HBTRC-MLC Human Prefrontal Cortex Agilent (Jun11) mlratio.” For the Liang data set, cases include ~16 normal aged subjects and ~16 AD subjects (Liang et al., 2006, 2008), and gene expression was profiled for six regions on the Affymetrix platform. For the Meyers data set, expression was profiled for temporal cortex and cortical tissue from 187 normal aged adults and 176 AD cases on the Illumina Sentrix Bead array (HumanRef-8) using Illumina's rank invariant transform (Webster et al., 2009). The HBTRC data set includes expression profiles for prefrontal cortex from ~170 normal subjects, ~230 HD subjects, and ~400 AD subjects using a custom-made Agilent 44K microarray. The Harvard Brain dataset was contributed by Merk Pharmaceutical through the Sage Bionetworks Repository. Additional information for each data set is available at genenetwork.org.

### Covariation of miRNA biogenesis genes and central nervous system phenotypes

The BXD phenotype database was used to find the top 100 correlates of *Pasha, Drosha*, and *Dicer* expression in each brain region. The Spearman correlation coefficient was used to reduce the effect of outliers and a moderately strict criterion of significance of *P* < 0.01 was used to select the top correlates. In the case of multiple probe sets targeting different mRNA regions of the same gene, the highest expressed probe set was used. An adjusted *p*-value was calculated based on 20,000 permutations for the Central Nervous System (CNS) phenotype correlations to address the large number of phenotypes available in GeneNetwork. The adjusted *p*-value was used to sort and prioritize CNS phenotypes. The criterion for determining a significant correlation was an adjusted *p*-value less than 0.01 after permutation tests (Table S3) or a *p*-value less than 0.005 (Table S2).

### miRNA biogenesis gene selection and network construction

Mouse genes with the following Gene Ontology (GO) categories—primary miRNA processing (GO:0031053), pre-miRNA processing (GO:0031054), and production of miRNAs involved in gene silencing by miRNA (GO:0035196)—were selected using Amigo (amigo.geneontology.org). Literature searches were used to confirm, expand, and refine categories. Unique gene symbols were used to retrieve expression data from each GeneNetwork data set. Probe sets targeting each gene were screened based on specificity (probe sets target the gene of interest and do not overlap polymorphisms that affect hybridization (see Ciobanu et al., 2010 for review), expression (generally greater than 8 log2 expression units), and target [probe sets targeting exons have less complicated expression patterns and network correlations compared to probe sets targeting 3′ UTR regions, see (Mulligan et al., 2012) for review]. All correlations, network graphs, and principal component analyses (PCA) were performed using tools and functions available in GeneNetwork. Briefly, PCA is implemented using custom Python code and is automatically computed for correlation matrices with three or more members. Output of the PCA function are a list of trait vectors representing the top principal components that can be used with any GeneNetwork tool, a Scree Plot, and a Factor Loading Plot.

### Expression of miRNA biogenesis genes after ethanol exposure in BXD strains

We used expression data collected from the prefrontal cortex, ventral tegmental area, and nucleus accumbens of BXD strains 4 h following either a saline injection (the “VCU Sal” data sets) or a 1.8 g/kg interperitoneal injection of ethanol (“VCU EtOH” data sets) (Kerns et al., 2005). Biological response to ethanol without accounting for the effect of genotype was measured by an uncorrected paired *t*-test for each region between saline and ethanol treatment groups. A more sophisticated analysis (Fisher's Exact Test) that takes differential response to treatment due to the effect of genotype into account has been described previously for this data set (Wolen et al., 2012) and genes significantly regulated by ethanol treatment are listed in Table S1 of that publication. We queried this table for alcohol responsive miRNA biogenesis genes.

## Results

### Expression and genetic regulation of miRNA biogenesis genes in mouse brain

*Drosha, Pasha*, and *Dicer* expression is measured from multiple probe sets that target different mRNA features. Probe sets that target coding exons and the 3′ UTR are well-expressed across the amygdala, prefrontal cortex, striatum, and hippocampus (>8) while probe sets targeting introns are generally not well-expressed (<6) and are excluded from future analysis (Table 1). An exception occurs for the last four coding exons of *Pasha* (1422981_at), which are well-expressed in the hippocampus but not in the prefrontal cortex and striatum. Probe sets do not show a strong bias in expression measurements caused by SNPs or other variants overlapping the probe target sequence and thus, are appropriate for use in further analysis.

**Table 1:**
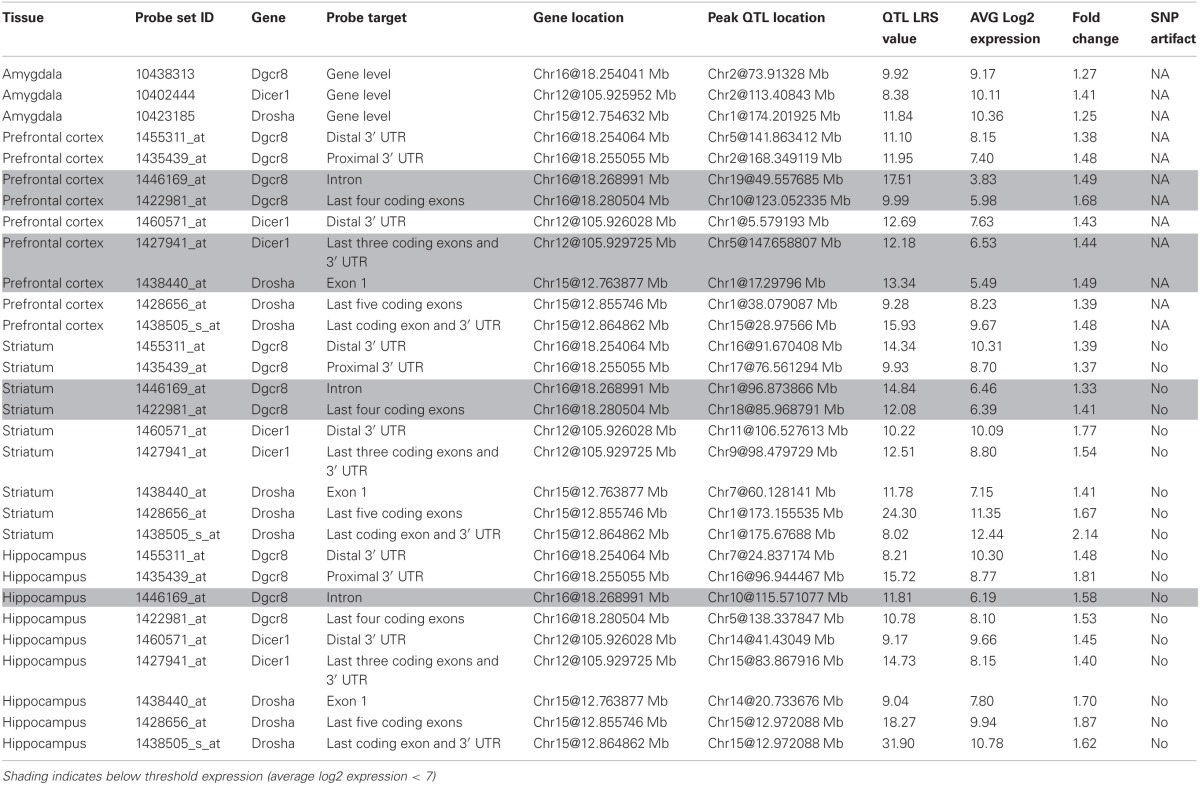
**Expression of key miRNA biogenesis genes across brain regions**.

Shading indicates below threshold expression (average log2 expression < 7)

**Figure 1:**
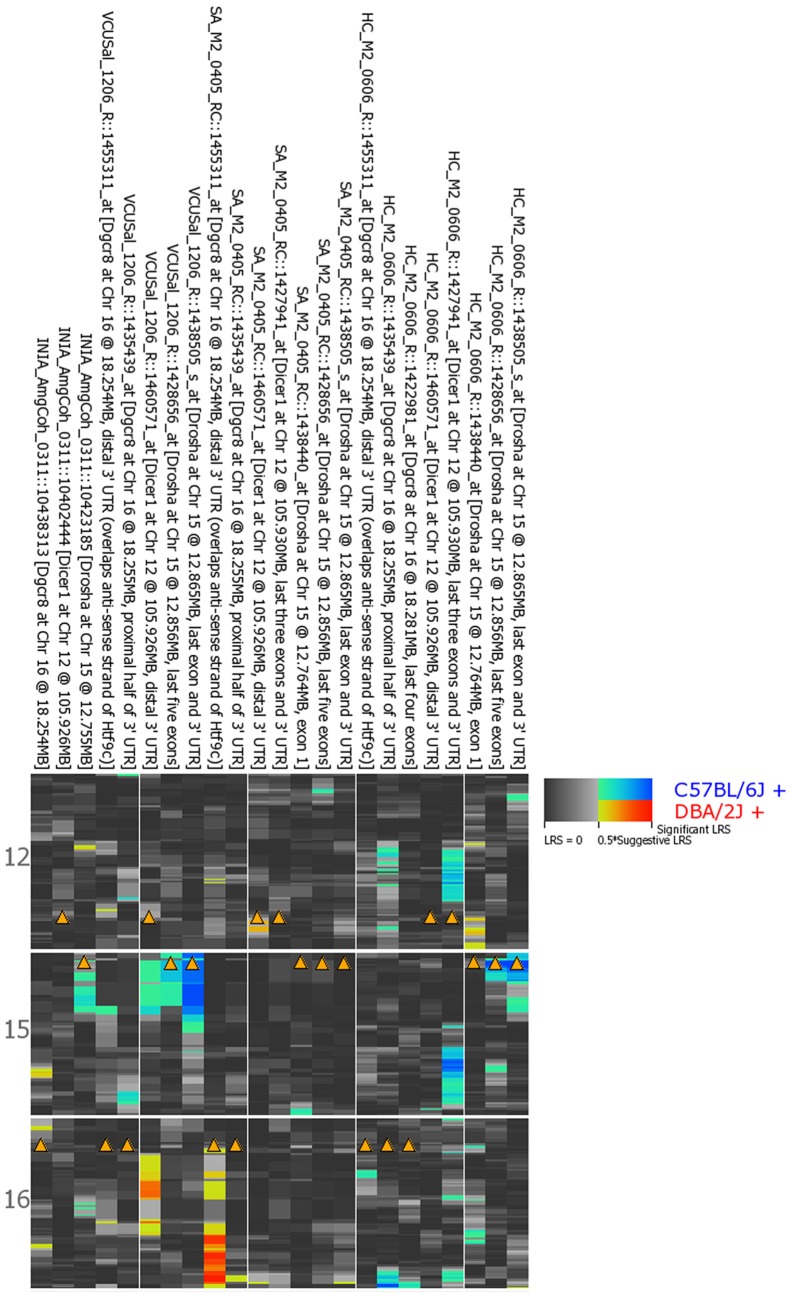
**QTL heat map for *Dicer, Drosha*, and *Pasha* across brain regions from the BXD population**. Warm colors indicate higher expression from the D2 allele and cooler colors indicate higher expression associated with inheritance of the B6 allele. Arrowheads indicate genomic location of each miRNA biogenesis gene. A limited number of *Drosha* probe sets are associated with significant (hippocampus) or suggestive (prefrontal cortex) regulation of expression by local sequence variants—a *cis* eQTL. Both probe sets have higher expression in BXD strains that inherited the B6 allele at this locus. INIA_AmgCoh, Amygdala; VCUSal, Prefontal Cortex; SA, Striatum; HC, Hippocampus.

Variation among the BXD family of strains is generally modest (<1.4) with the exception of *Drosha* expression in the striatum and hippocampus and *Pasha* expression in the hippocampus (~2 fold). None of the biogenesis genes show consistent and significant local modulation of expression by sequence variants located within or near each gene locus (a *cis* eQTL) across multiple probe sets and brain regions (Figure 1). However, expression of the last few exons and the 3′ UTR of *Drosha* in the hippocampus are regulated by a significant *cis* eQTL and expression of the last exon and 3′ UTR is regulated by a suggestive *cis* eQTL in the prefrontal cortex. Each biogenesis gene is genetically uncoupled from the others such that the expression of *Pasha, Dicer*, or *Drosha* is not modulated from the physical location of the other two genes (a *trans* eQTL) (Figure 1). These results suggest that, with the exception of possible 3′ UTR or splice isoforms of *Drosha*, expression of miRNA biogenesis genes in the BXD population is not controlled by sequence variants in cis-regulatory regions but is likely the result of many complex genetic interactions of small effect size.

### Coexpression of Pasha, Dicer, and Drosha

**Figure A1:**
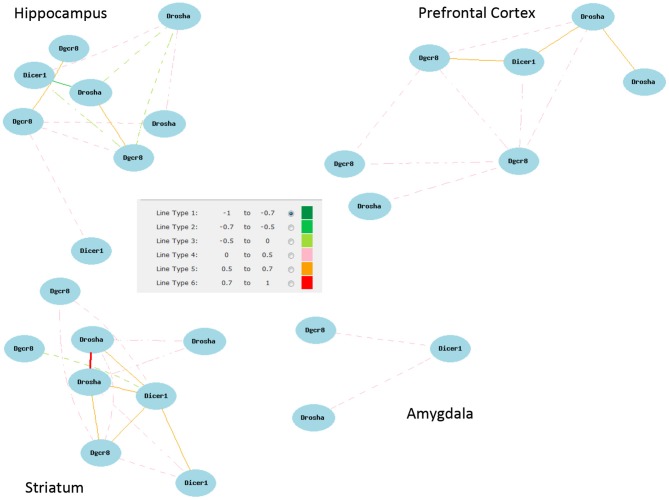
**Network correlations for all *Dicer, Pasha*, and *Drosha* (*Dgcr8*) probe sets**. Correlations shown are for all probe sets with average log 2 expression greater than 7 from each brain region data set.

**Figure A2:**
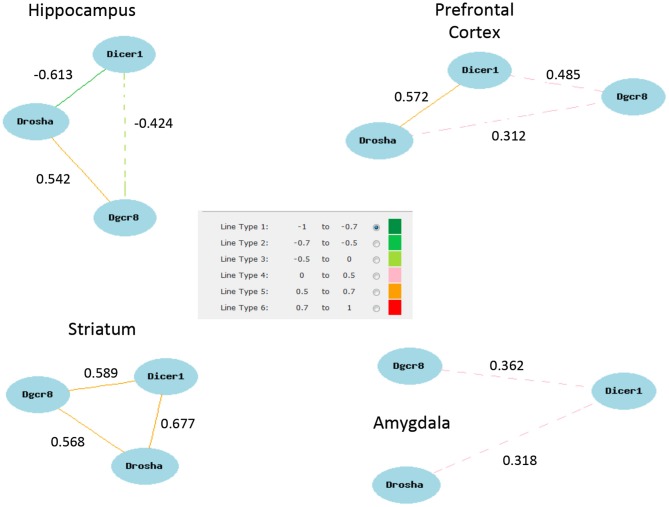
**Network correlations for *Dicer, Pasha*, and *Drosha* (*Dgcr8*)**. Correlations shown are for the highest expressing probe sets from each brain region data set. The exception is the hippocampus for which the highest pairwise correlations among all probe sets were selected (1422981_at, 1460571_at, 1438440_at).

Biogenesis genes belong to the same biological pathway and expression of these key genes may be coordinately regulated. Indeed, *Pasha, Drosha*, and *Dicer* expression in multiple brain regions tends to be tightly correlated (Figures A1, A2). Average network correlations are 0.6 (*p* < 0.01), 0.5 (*p* < 0.01), 0.45 (*p* < 0.01), and 0.3 (*p* < 0.05) for the striatum, hippocampus, prefrontal cortex, and amygdala, respectively. Network complexity arises from the existence of multiple probe sets targeting different mRNA regions of the same gene (Figure A1). This is typical for Affymetrix M430 arrays and probe sets (Mulligan et al., 2012). With the exception of the hippocampus, networks constructed from the three key biogenesis genes using the highest expressing probe sets tend to be positively correlated. Coexpression of these three genes in each brain region cannot be explained by a single genetic locus, yet another indication of complex network control of expression of miRNA biogenesis genes.

### The striatal miRNA biogenesis network

**Figure 2:**
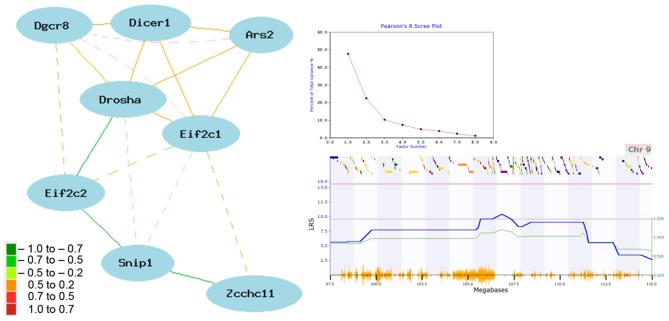
**Network correlations for miRNA biogenesis genes in the striatum**. The network shown in the left panel was constructed using the highest expressing probe sets from the striatum data set [HBP Rosen Striatum M430V2 (Apr05) RMA Clean]. Correlation strength is indicated by network edge color with warm colors indicating positive correlations and cool colors indicating negative correlations. Common aliases: *Eif2c1* (*Ago1*), *Eif2c2* (*Ago2*), *Zcchc11* (*Tut4*), and *Ars2* (*Srrt*). Results of a principal component analysis are shown in the top right box. Principal components or loading factors are shown on the X-axis and percentage of variance explained is shown on the Y-axis. A suggestive eQTL on Chr9 controls the expression of principal component 1 (lower right box). The blue line plots the LRS value by physical position on Chr9. The actual LRS value is shown on the left Y-axis and the additive effect is shown on the right Y-axis. Yellow tick marks on the bottom X-axis show position of SNPs and colored bars on the upper X-axis show positions of genes. Red and gray horizontal lines mark the threshold for genome-wide significant (*P* < 0.05) and suggestive thresholds based on 2000 permutations.

The striatum is a key region implicated in many CNS diseases and disorders, including psychiatric disease and addiction. It is also composed largely of a single cell type—spiny projection neurons—making it an excellent tissue for exploring the network connectivity of miRNA biogenesis genes. Eight genes involved in this pathway are connected within a single striatal expression network by at least two nodes with a |*r*| > 0.30 (Figure 2). *Pasha* (*Dgcr8;* 1455311_at), *Dicer1* (1460571_at), *Drosha* (1438505_s_at), *Ars2 (Srrt;* 1417655_at), and *Ago1* (*Eif2c1*; 1434331_at) are well-connected and positively correlated. In contrast, *Ago2* (*Eif2c2*; 1435636_at) and *Zcchc11* (*Tut4*; 1437395_at) are negatively correlated with network members. *Snip1* (1459773_x_at) membership in the network is more complex, with both negative and positive correlations with other members. In general, positive and negative correlations are consistent with known regulatory interaction between these eight genes. For example, *Snip1* and *Ars2* are accessory proteins involved in pri-miRNA processing that act as positive regulators of Drosha-mediated processing in the canonical pathway (Krol et al., 2010). *Ago1* and *Ago2* are accessory proteins for the RISC and it is less clear why *Ago2* would be negatively correlated with network members (Krol et al., 2010). *Zcchc11* acts a suppressor to miRNA biogenesis (Heo et al., 2009).

A tightly correlated biological network can be reduced to an expression signature—or principal component—using principal component analysis (PCA). For the striatal miRNA biogenesis network we used the first principal component (PC1) as a network signature to identify phenotypes that covary with network expression. PC1 for the miRNA biogenesis network in the striatum explains ~50% of the variance in network coexpression (Figure 2). PC1 expression maps to a region on Chr 9 (105.67–106.32 Mb) with a LRS of 9.6, which meets the genome-wide suggestive threshold (Figure 2). The top behavioral phenotypes correlated with the expression of PC1 (*p* < 0.005) are all psychiatric- or addiction-related traits (Table S1). This includes anxiety (12463, 12476, 12381, 12460, 12478, 12464, 12462, 12383) and sensorimotor gating traits (11942) and locomotor responses to cocaine (10488), ethanol (11701), and morphine (11342, 11856, 11343). Taken together these results suggest a role for miRNA biogenesis genes and miRNA pathways in psychiatric- and addiction-related phenotypes.

### Covariation with addiction and behavioral phenotypes

**Figure 3:**
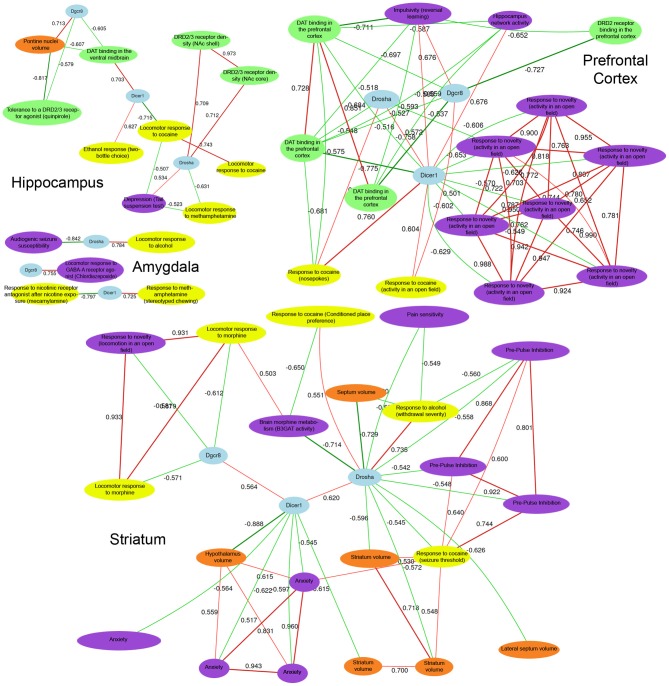
**CNS phenotypes strongly correlated with miRNA biogenesis brain gene expression**. Detailed phenotype descriptions can be found in Table S2 and Table S3. Top correlated phenotypes were selected for Hippocampus, Amygdala, Prefrontal Cortex, and Striatum based on permutation testing (adjusted *P*-value < 0.01) and a network was constructed for each region using tools in GeneNetwork. Negative network correlations are indicated by cool line colors and positive correlations are indicated by warm line colors. Correlation threshold is *r* = 0.5. At the center of each network, *Pahsa (Dgcr8), Drosha*, and *Dicer* nodes are shaded in blue. Yellow, purple, green, and red nodes indicate addiction-, psychiatric-, dopaminergic-, and morphology-related traits, respectively. In each brain region, with the exception of the amygdala, many behavioral phenotypes related to addiction and psychiatric disease are highly correlated with the expression of miRNA biogenesis genes. Importantly, dopaminergic phenotypes and morphological phenotypes in key brain regions that play a role in addiction and psychiatric disease processes are also correlated with behavioral phenotypes and expression of miRNA biogenesis genes. This indicates an overlap between miRNA biogenesis gene expression and developmental processes and biological pathways involved in addiction and psychiatric disease-related phenotypes.

Expression of each miRNA biogenesis gene is significantly correlated with many central nervous system phenotypes in the amygdala, striatum, prefrontal cortex, and the hippocampus (Figure 3 and Tables S2, S3). These brain regions are important for reward, decision-making, and emotional response. Alterations in gene expression and neuronal circuitry in these regions have been implicated in psychiatric disorders and addiction. To better assess the involvement of *Drosha, Dicer*, and *Pasha* in these disorders, we examined the correlation between expression of each biogenesis gene and the ~1500 BXD CNS phenotypes available at the GeneNetwork web resource. As shown in Figure 3 for each brain region, the expression of miRNA biogenesis genes is highly significantly correlated with addiction-related phenotypes. The expression of *Dicer* and *Drosha* is correlated with cocaine, ethanol, and methamphetamine responses. *Pasha* is correlated with endophenotypes relevant to drug taking behaviors, such as a measure of impulsivity and response to a novel environment.

The dopamine system has been strongly linked to addiction processes, particularly the reward aspects of addiction (Wise, 2009) and all of the miRNA biogenesis genes exhibit strong correlations with various phenotypes related to the dopamine system across most of the brain regions surveyed. For example, *Pasha* and *Dicer* expression in the hippocampus is correlated with response to a dopamine D2-type (Drd2) and D3-type receptor agonist (10048) and dopamine transporter levels in the ventral midbrain (10282), respectively. Prefrontal cortical expression of *Dicer* and *Pasha* is correlated with DRD2 and dopamine transporter levels in the same tissue (10280 for *Dicer*; and 10267, 10279, 10281 for *Pasha*). Additionally, the expression of *Dicer* and *Drosha* in the striatum is correlated with striatal volume (13440 for *Dicer* and 11498 and 10998 for *Drosha*) suggesting a possible morphological link.

The expression of miRNA biogenesis genes is also correlated with endophenotypes for psychiatric disorders. For example, *Drosha* expression in the hippocampus is correlated with measures of depression (12554). Prefrontal cortical expression of *Pasha* is correlated with a measure of impulsivity (12731) and *Dicer* levels in the striatum are correlated with anxiety measurements (12477, 12478, 12451, 12476). Additionally, striatal levels of *Drosha* are correlated with pre-pulse inhibition of the acoustic startle response (11942, 11684, 11941) which is a measure of sensorimotor gating—a process disrupted in many schizophrenic patients. Our results show that the expression of miRNA biogenesis genes is directly related to numerous phenotypes associated with addiction and psychiatric disease as well as other molecular pathways linked to these phenotypes.

### Expression of miRNA biogenesis genes after ethanol exposure in BXD strains

To directly assess whether exposure to ethanol alters the expression or genetic regulation of miRNA biogenesis genes, we queried expression data sets collected from the prefrontal cortex, ventral tegmental area, and nucleus accumbens of BXD strains four hours following either a saline injection or a 1.8 g/kg interperitoneal injection of ethanol (Kerns et al., 2005). A significant expression difference between treatment groups—without accounting for genotype—was detected by paired *t*-test (uncorrected, *p* < 0.05) in the nucleus accumbens for *Pasha* probe sets targeting the 3′ UTR (1435439_at) and an intron with low expression (1446169_at). A recent analysis allowing for differential response to ethanol due to genetic variation among the BXD strains identified *Ago2* (1426366_at) and *Pasha* (1455311_at) probe sets in the prefrontal cortex and *Ars2* (1417655_a_at) and *Ago2* (1426366_at) probe sets in the nucleus accumbens as being regulated by ethanol (Wolen et al., 2012). Significant *cis* modulation of expression (LRS = 16.2) for a *Pasha* probe set (1455311_at) targeting the distal 3′ UTR was also detected after ethanol treatment—but was absent in saline injected controls—in the nucleus accumbens. Exposure to alcohol significantly alters the genetic modulation of *Dicer* (1427941_at; Chr9@47.04 Mb; LRS = 16.97) in the ventral tegmental area and both *Dicer* (1460571_at; Chr9@69.81 Mb; LRS = 15.6) and *Drosha* (1438505_s_at, Chr9@65.31 Mb; LRS = 15.7) in the prefrontal cortex. As observed in other naïve BXD brain expression data sets, no significant modulation of expression was observed in control BXD strains. These results suggest that miRNA biogenesis gene expression can be modulated by exposure to alcohol.

### Expression of miRNA biogenesis genes in human brain

**Figure A3:**
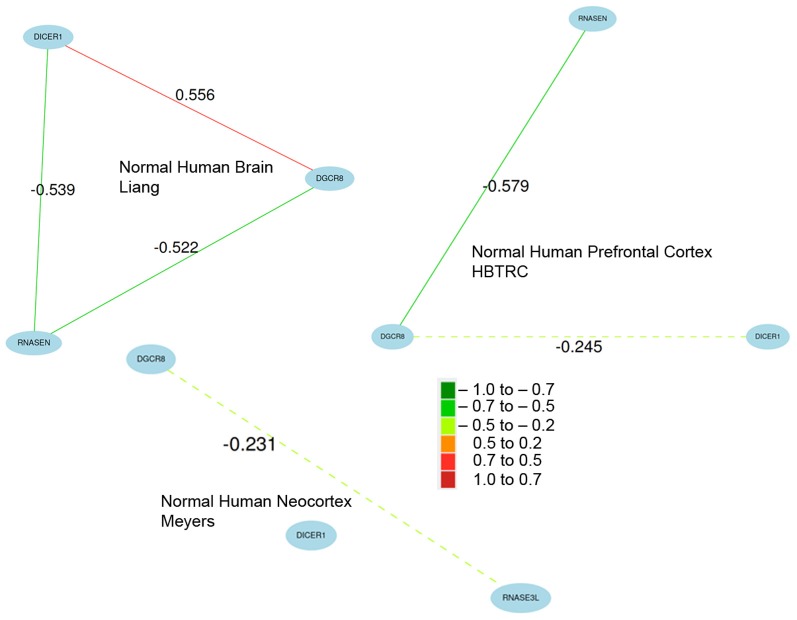
**Coexpression of key miRNA biogenesis genes in normal human brain data sets**. Network correlations are especially strong across multiple brain expression data sets from the study by Liang and colleagues (2006, 2008). Network connections are less robust in the data sets contributed by Meyers and colleagues (Webster et al., 2009). Because of the large number of variants and differences in probe design across platforms, probe sets were chosen based on expression and overall network connectivity.

Expression levels of miRNA biogenesis genes—*DICER1, DGCR8* (*PASHA*), and *DROSHA* (*RNASEN or RNASE3L*) show a remarkable level of variation across normal aged human brain (~1.3–68 fold difference) using different platforms, populations, and probe sets (Table S4). Human populations are incredibly polymorphic and some of this variation reflects true expression differences as well as technical artifacts due to genetic variants that overlap the probe target sequence and alter binding. In many of the human data sets, there is considerable covariation of *PASHA, DROSHA*, and *DICER1* (Figure A3).

**Figure 4:**
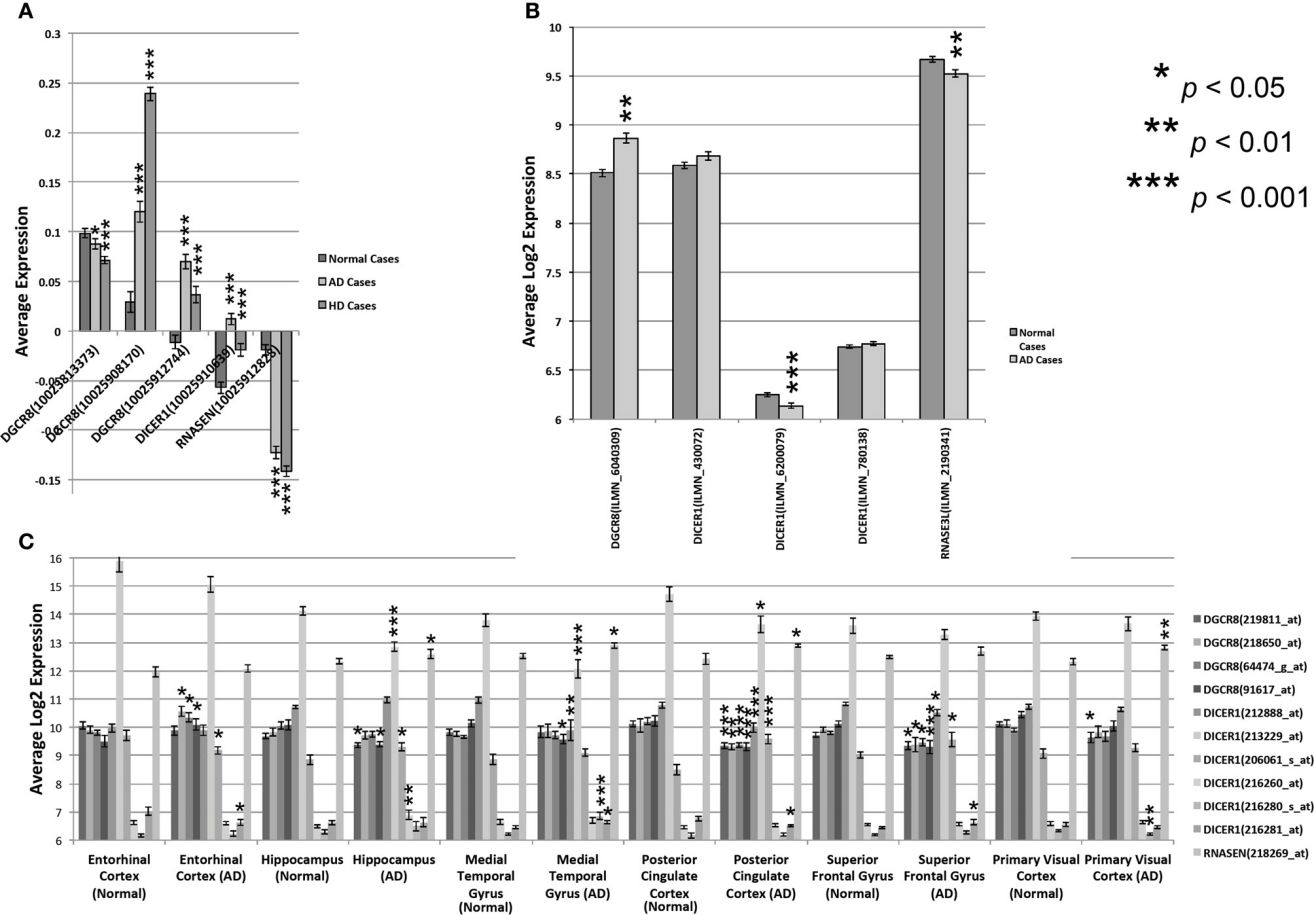
**Expression of miRNA biogenesis genes across human cases**. Expression of multiple *DICER, DROSHA*, and *PASHA* probe sets from different regions of normal and diseased human brain tissue. **(A)** Expression levels are shown for the three miRNA biogenesis genes in the prefrontal cortex of normal or Alzheimer (AD) or Huntington Disease (HD) cases (HBTRC data). The average expression ratio of each gene in normal, AD, and HD cases compared to a common reference sample is shown on the Y-axis. **(B)** Average log2 expression (Y-axis) of *DICER, PASHA*, and *DROSHA* is shown for the Neocortex of normal or AD patients. Expression data was standardized to have an average log2 expression of 8 with a standard deviation of ± 2. **(C)** Average log2 expression of all three genes, as assayed by different probe sets, is shown on the Y-axis for 6 different brain regions from normal and AD cases. Expression data was standardized to have an average log2 expression of 8 with a standard deviation of ± 2. Significant expression differences are often observed for at least one probe set between normal and Alzheimer or Huntington Disease cases. Significance was determined by Student's two-tailed *t*-test.

To assess the involvement of miRNAs and miRNA biogenesis genes in human neurological diseases, we explored the expression of these genes across several brain regions from control and Alzheimer's (AD) and Huntington's (HD) disease cases. AD is the major cause of adult dementia and involves early loss of neurons—especially cholinergic—in the cortex and hippocampus. AD is also associated with a wide range of psychiatric symptoms (Koppel et al., 2012). HD involves the selective loss of striatal populations of medium spiny projection neurons and concomitant alterations in mood—often depression—and motor coordination. Intriguingly, we found highly statistically significant expression differences for miRNA biogenesis genes between control and AD or HD disease cases (Figure 4 and Table S4) based on uncorrected *t*-tests between control and disease cases. For HD cases compared to controls, most *PASHA* (*DGCR8*) probe sets and *DICER1* have higher expression in the prefrontal cortex while *DROSHA* expression is decreased. For AD cases compared to controls, there is generally significantly (*p* < 0.05) higher expression of *PASHA* in prefrontal cortex, neocortex, and entorhinal cortex and significantly lower expression in the hippocampus, medial temporal gyrus, cingulate cortex, and superior frontal gyrus. Significantly lower expression of most *DICER* probe sets (212888_at, 213229_at) is detected in AD cases compared to controls and significantly lower expression of *DROSHA* in AD cases compared to controls is also observed in most data sets (HBTR and Meyers) (Figure 4). Taken together, this survey of miRNA biogenesis gene expression across brains regions from normal or disease cases suggests an association between expression of these genes and AD and HD. However, the mechanism underlying this relationship and whether or not the observed alterations in miRNA biogenesis gene expression are causal or downstream of the disease state are not known.

## Discussion

In this study, we used bioinformatic and genetic analyses of miRNA biogenesis genes to show that microRNAs could be critical modulators of pathways that underlie differential responses to ethanol or drugs of abuse and differential indicators of psychiatric disease. Our initial analysis examined the expression of these genes and showed that there were both strain-specific and brain region-specific differences. This wide range of variation in expression was found in both mouse and human populations. Despite this variation, the key components of the pathway—*Drosha, Pasha*, and *Dicer*—are generally strongly correlated across brain regions. Subsequent analyses were used to assess whether these expression differences could impact phenotypes. For example, *Dicer, Drosha*, and *Pasha* are correlated with key CNS phenotypes related to addiction and psychiatric disorders. Additionally, exposure to alcohol alters the expression and genetic regulation of these genes suggesting that differential responses between strains as well as a priori expression differences across strains could be critical in mediating phenotypic differences.

We observed a large level of variation in the expression of *Drosha* and *Pasha* in the striatum and hippocampus. Using the BXD genetic mouse resource we can rule out technical sources of variation and focus on possible mechanisms of genetic regulation. Interestingly, none of these genes are consistently modulated by local sequence variants that influence expression, at least in brain. It also appears that there is no single *trans* modulatory locus that controls a major component of their expression. Even in the striatum, where there is a relatively homogenous population of cells and strong correlations among eight miRNA biogenesis pathway members, the best single locus modulating network expression (Chr 9 from 105.67 to 106.32 Mb) explains less than 50% of the joint variation in expression. Candidate genes within this region—*Atp2c1, Tlr9*, and *Alas1*—may have a broad, yet subtle, influence on miRNA biogenesis pathways. Indeed, many genes and steps are involved in the production of miRNA and the process is tightly regulated. A major mutation in this pathway severely compromises organism function very early in development. The BXD genetic reference population is not segregating for a major mutation in key miRNA biogenesis genes, but proves useful to assess how subtle variation influences their expression and behavioral phenotypes.

Across brain regions and platforms, expression of *Pasha, Dicer*, and *Drosha* significantly covaries with the expression of multiple addiction phenotypes and psychiatric-related traits. Both *Dicer* and *Drosha* were correlated with the response to cocaine, ethanol, and methamphetamine. This suggests that these genes are related to the behavioral response to a wide-range of substances and are more related to addiction, *per se*, rather than to any specific substances. In addition, the category or type of response shows considerable consistency. For example, locomotor activities are correlated with expression of all three enzymes in multiple brain regions and to multiple substances. This suggests that certain responses may have a stronger relationship to the expression of the miRNA biogenesis genes. In addition, the expression and genetic modulation of *Pasha* in the nucleus accumbens is significantly altered after ethanol exposure in BXD strains. The same exposure to alcohol results in *trans* modulation of *Dicer* and *Drosha* by loci on Chr9 at 47.04, 65.31, and 69.81 Mb in the ventral tegmental area and the prefrontal cortex. A large region on Chr9 (~37 Mb to 99 Mb) has previously been associated with alcohol preference (*Alpq3, Ap5q, Alpq1*) and consumption (*Etohc3*) in B6D2-derived populations, including BXD strains (Phillips et al., 1994, 1998; Belknap et al., 1997; Tarantino et al., 1998; Belknap and Atkins, 2001; Kerns et al., 2005; Bice et al., 2006, 2008, 2009; Mulligan et al., 2006; Weng et al., 2009). The alcohol QTL region on Chr 9 in mouse is syntenic to a region on human Chr15 (Ehlers et al., 2010) and association studies in human populations have identified alcohol dependence QTLs within this region of synteny at 50–53 Mb (Ehlers et al., 2004). These results indicate that alcohol exposure changes the expression and genetic regulation of miRNA biogenesis genes through an unknown mechanism. This mechanism may influence human alcohol dependence as well.

Intriguingly, all three key biogenesis genes are also correlated with behavioral measures of depression, anxiety, and schizophrenia. There is high comorbidity between psychiatric disease and addiction (Swendsen et al., 2010) suggesting that these disorders involve similar brain pathways. Although the exact mechanism explaining the correlation cannot be addressed in this analysis, the strong covariation supports the involvement of miRNA biogenesis genes in the expression of these phenotypes and warrants deeper scrutiny.

The striatum is a brain region important in motivation, decision, and reward as well as addiction, psychiatric, and mood disorders. We observed a strong correlation between expression of key members of the dopamine pathway and miRNA biogenesis gene expression in most brain regions. The strongest expression signature from eight correlated biogenesis genes in the striatum was strongly and significantly associated with locomotor responses to cocaine, ethanol, and morphine as well as anxiety and sensorimotor gating traits. miRNA and miRNA biogenesis genes have been previously linked to maintenance of the reward pathway and psychiatric disease and may have an exquisite interaction with neuronal function in the striatum. For example, loss of Dicer in dopaminergic neurons and Drd2 expressing medium spiny neurons leads to death of those neuronal populations (Kim et al., 2007; Davis et al., 2008). Loss of *Ago2* in adult medium spiny neurons expressing Drd2 rendered mice insensitive to cocaine and they failed to develop dependence (Schaefer et al., 2010). Alcohol consumption in adult mice leads to an approximately 2-fold increase in the level of miR-9 in the striatum followed by a reduction in expression of a subset of mRNA isoforms that contain miR-9 targets in the 3′ UTR and code for a large conductance calcium and voltage gated potassium channel (Pietrzykowski et al., 2008). In human populations, microdeletions that include the DGCR8 gene (PASHA) cause DiGeorge syndrome—a multispectrum disorder associated with a high risk of schizophrenia. We are still exploring the role of miRNA in adult brain but it may be that this class of regulatory molecules and their product miRNAs are functionally more critical in certain regions and cell types then others.

Our analysis of miRNA biogenesis gene expression and regulation was primarily based on extensive data collected from the BXD genetic mouse population. The majority of our findings in mouse also extend to human brain tissue where we observed an even greater level of expression variation and coexpression of *PASHA, DICER*, and *DROSHA*. These genes are highly polymorphic in humans and without genotyping we cannot eliminate the possibility that expression variation is caused by technical artifacts related to probe design and probe target sequence. Interestingly, we also found highly statistically significant expression differences for miRNA biogenesis genes between control and Alzheimer's (AD) and Huntington's (HD) disease cases (Figure 4). It is highly unlikely that SNPs and other variants overlapping probe sets could cause this pattern of miRNA biogenesis gene expression between control and disease cases. These differences also suggest that miRNA processes play some role in the progression of HD and AD, either as drivers or as downstream consequences. Indeed, down-regulation of several miRNAs has been documented in HD (Johnson et al., 2008; Packer et al., 2008), which features early loss of medium spiny neuronal cell populations in the striatum. However, little is known about the involvement of miRNAs and miRNA biogenesis genes in AD. Our results provide additional support for the relevance of these genes in adult human brain and are further impetus for examining their role in neurological disease, addiction, and psychiatric disorders.

Taken together, our study provides evidence for the role of miRNA biogenesis genes and pathways in addiction- and psychiatric-related disease processes and suggests that miRNA pathways may be perturbed in human brain degenerative disorders such as HD and AD. Finally, our analysis provides additional support for an exquisite and critical role between miRNA and miRNA biogenesis and the function of the striatum. Most of our analysis is based upon correlations, which do not necessarily indicate direct causal relationships. The next step will be to establish causal links between the expression of miRNA and key biogenesis genes and these important CNS phenotypes and diseases.

### Conflict of interest statement

The authors declare that the research was conducted in the absence of any commercial or financial relationships that could be construed as a potential conflict of interest.
